# Influenza Virus Infection Induces ZBP1 Expression and Necroptosis in Mouse Lungs

**DOI:** 10.3389/fcimb.2019.00286

**Published:** 2019-08-07

**Authors:** Yun Wang, Qin Hao, Jon M. Florence, Bock-Gie Jung, Anna K. Kurdowska, Buka Samten, Steven Idell, Hua Tang

**Affiliations:** ^1^Department of Cellular and Molecular Biology, The University of Texas Health Science Center at Tyler, Tyler, TX, United States; ^2^Department of Pulmonary Immunology, The University of Texas Health Science Center at Tyler, Tyler, TX, United States; ^3^Texas Lung Injury Institute, The University of Texas Health Science Center at Tyler, Tyler, TX, United States

**Keywords:** Influenza A virus, ZBP1, programmed cell death, necroptosis, lung

## Abstract

Programmed cell death and especially necroptosis, a programmed and regulated form of necrosis, have been recently implicated in the progression and outcomes of influenza in mouse models. Moreover, Z-DNA/RNA binding protein 1 (ZBP1) has been identified as a key signaling molecule for necroptosis induced by Influenza A virus (IAV). Direct evidence of IAV-induced necroptosis has not been shown in infected lungs *in vivo*. It is also unclear as to what cell types undergo necroptosis during pulmonary IAV infection and whether ZBP1 expression can be regulated by inflammatory mediators. In this study, we found that IAV infection induced ZBP1 expression in mouse lungs. We identified that mediators implicated in the pathogenesis of IAV infection including interferons (IFNs), TNFα, and agonists for Toll-like receptors 3 and 4 were potent inducers of ZBP1 expression in primary murine alveolar epithelial cells, bone marrow derived macrophages, and dendritic cells. We further found that IAV infection induced a strong necroptosis through phosphorylation of the necroptosis effector mixed lineage kinase domain-like protein in infiltrating immune cells and alveolar epithelial cells by day 7 post-infection. Lastly, we found different cell type-specific responses to IAV-induced cell death upon inhibition of caspases and/or necroptosis pathways. Our findings provide direct evidence that IAV infection induces necroptosis in mouse lungs, which may involve local induction of ZBP1 and different programmed cell death signaling mechanisms in alveolar epithelial and infiltrating inflammatory cells in the lungs.

## Introduction

Influenza is a highly contagious, acute respiratory disease that can promote exacerbations of airway and lung disorders as well as cardiovascular diseases (Estabragh and Mamas, 2013; Michael et al., 2013; Short et al., 2014). Influenza A virus (IAV) targets lung epithelial cells and induce host immune responses, causing annual epidemics and every 10–50 years, pandemics of variable severity. Influenza affects all age groups, results in considerable morbidity and mortality, and exacts a formidable toll on world health and economics. For example, the influenza outbreak in the United States during the 2017–2018 season caused illness in millions of people and an estimated 959,000 hospitalizations and 79,400 deaths from influenza according to the Center for Disease Control (www.cdc.gov/flu/about/burden/2017-2018.htm). This incidence was higher than in any season since the 2009 pandemic. Hence influenza poses a continuing and substantive threat to human health in the US.

Programmed cell death and especially necroptosis, a programmed and regulated form of necrosis, has been recently implicated in the disease progression and outcomes of influenza in murine models (Rodrigue-Gervais et al., 2014; Kuriakose et al., 2016; Nogusa et al., 2016; Thapa et al., 2016; Downey et al., 2017; Xu et al., 2017). The central model of necroptosis is executed by receptor-interacting protein kinase-3 (RIPK3) and mixed lineage kinase domain-like protein (MLKL) (Cho et al., 2009; He et al., 2009; Sun et al., 2012; Zhao et al., 2012; Wang et al., 2014). Recent studies in murine models have identified a protein called Z-DNA/RNA binding protein 1 (ZBP1; also known as DAI/DLM-1) as a key mediator of IAV-induced programmed cell death including apoptosis, necroptosis, and pyroptosis through its interaction with RIPK3, thus controlling the progression of influenza and animal survival (Kuriakose et al., 2016; Thapa et al., 2016; Kesavardhana et al., 2017; Kuriakose and Kanneganti, 2018). Necroptosis can also be induced by the activation of tumor necrosis factor (TNF) family of death receptors, Toll-like receptors (TLR) 3 and 4 through RIPK1-RIPK3-MLKL or TRIF-RIPK3-MLKL pathways, respectively (Kaiser et al., 2013; Silke et al., 2015; Wallach et al., 2016; He and Wang, 2018). Necroptosis induced by the activation of death receptors and TLR3 contributes to host cell death as IAV infection normally induces the production of TNF superfamily death ligands and double-stranded RNA (dsRNA) (Yoo et al., 2013; Son et al., 2015). Despite the critical role of necroptosis in the pathogenesis of influenza, direct evidence of IAV-induced necroptosis has not been shown in infected mouse lungs *in vivo*. The cell types that predominantly undergo necroptosis during IAV infection in infected lungs are likewise poorly understood. Moreover, it is not clear whether ZBP1 expression can be regulated by inflammatory mediators in addition to IAV. This study was designed to address these gaps in current knowledge.

## Materials and Methods

### Antibodies and Reagents

The specific antibody against murine phosphorylated-MLKL at Ser345 (clone7C6.1, no. MABC1158), RIPK1 inhibitor II (Nec-1s, no. 504297), and RIPK3 inhibitor GSK872 (no. 530389) were from EMD Millipore (Burlington, MA). IAV nucleoprotein (NP) antibody was from GeneTex (Irvine, CA). Mouse specific ZBP1 antibody (no. AG-20B-0010) was from AdipoGen (San Diego, CA). Actin (no. A4700) and vinculin (no. V9131) antibodies and LPS (*Escherichia coli* 0111:B4) were from Sigma (St. Louis, MO). Alexa Fluor-488 anti-mouse CD45 (no. 103121), FITC anti-mouse CD192 (CCR2, no. 150607), and TruStain fcX (anti-mouse CD16/32, no. 101319) antibodies, and recombinant mouse interlulin-4 (IL-4, no.574304), macrophage-colony stimulating factor (M-CSF, no. 576404), and granulocyte/macrophage-colony stimulating factor (GM-CSF, no. 576304) were from Biolegend (San Diego, CA). Alexa Fluor-568 goat anti-mouse (no. A-11004) secondary antibodies, recombinant mouse IFNα2 (no. 14-8312) and IFNγ (no. 34-8311) were from Thermo Fisher Scientific (San Diego, CA). Polyinosinic-polycytidylic acid (poly(I:C)) (no. tlrl-pic) was from InvivoGen (San Diego, CA). Z-VAD-FMK (no. A1902) was from ApexBio (Boston, MA). Vector MOM immunodetection kit (PK-2200), Vector NovaRED substrate (SK-4800), Vector hematoxylin (H-3401), VectaMount permanent mounting medium (H-5000), and Vectashield hardset antifade mounting medium with DAPI (H-1500) were from Vector Laboratories (Burlingame, CA). Clarity Western ECL substrate was from Bio-Rad (Hercules, CA).

### Animal Experiments

All animal experiments were approved by the Institutional Animal Care and Use Committee at the University of Texas Health Science Center at Tyler. C57BL/6J mice were obtained from Jackson Laboratory (Bar Harbor, ME). Mice at 6–8 weeks old were anesthetized by intraperitoneal injection of ketamine (100 mg/kg) and xylazine (8.5 mg/kg) then administered either intranasal instillation of 50 μl sterile saline control vehicle or 50 μl saline containing 1,000 plaque-forming units (pfu) (about 2 LD_50_) H1N1 PR/8/34 (Charles River, Wilmington, MA). Mice were observed daily for signs of distress by monitoring general appearance, respiratory difficulties, body weight loss, and animal survival. Mice that lost more than 30% of their initial body weight were humanely euthanized by CO_2_ inhalation followed by cervical dislocation. On days 6 and 7 post infection, mice were euthanized and lungs were perfused and harvested. The whole lungs were fixed and used for histopathological analysis. In some cases, the lungs were inflated by intratracheal infusion of formalin. Formalin-preserved lungs were processed and embedded in paraffin via standard procedures. Lung homogenates were prepared in NP-40 lysis buffer (25 mM Tris-HCl, pH 7.5, 1% NP-40, 150 mM NaCl, 10 mM NaF, 1 mM phenylmethylsulfonyl fluoride, 10 μg/ml each of leupeptin, and aprotinin) for Western blots.

### Immunohistochemistry and Double Immunofluorescence Microscopy

Mouse lung sections (5 μm) were deparaffinized by incubation at 56°C for 30 min and subsequent xylene washes, then rehydrated by using a graded ethanol series. For antigen retrieval, the sections were incubated at 95°C for 10 min in 10 mM sodium citrate buffer (pH 6.0), then soaked in the buffer for additional 30 min at room temperature. The slides were then treated with 3% H_2_O_2_ for 5 min and washed in phosphate-buffered saline (PBS) with 0.1% Tween-20. Immunohistochemical staining was performed using MOM immunodetection kit according to the manufacturer's instructions (Vector Laboratories). Tissue sections were blocked and incubated overnight at 4°C with primary antibodies against ZBP1 (1:400) or p-MLKL (1:600). Non-immune normal isotype control antibodies served as negative controls. After incubation with biotinylated secondary antibody and ABC reagents, all the sections were incubated with peroxidase substrate Vector NovaRed for an equal amount of time to allow for suitable staining. The slides were counterstained with hematoxylin followed by a brief bluing, mounted with VectaMount medium, examined, and photographed using an Olympus BX41 microscope imaging system. Adobe Photoshop CS6 software was used for image processing.

For double immunofluorescence staining, lung tissue sections were blocked and incubated overnight at 4°C with p-MLKL (1:600) mouse monoclonal antibody, Alexa Fluor-488 conjugated CD45 or FITC conjugated CD192/CCR2 rat monoclonal antibodies (10 μg/ml each). Tissue sections were then washed with PBS containing 0.1% Tween-20 and incubated 25 min with Alexa Fluor-568 goat mouse secondary antibody (1:200) at room temperature in the dark. After washing, sections were mounted with Vectashield anti-fade mounting medium with DAPI and fluorescence was visualized and captured using a Nikon confocal imaging system. Adobe Photoshop CS6 software was again used for image processing.

### Primary Cell Preparation, Infection, and Treatment

Primary mouse alveolar epithelial cells (mAECs, C57-6053) were obtained from Cell Biologics (Chicago, IL), cultured in epithelial cell growth medium (M6621, Cell Biologics) and used for experiments within 3 passages. RAW264.7 murine macrophages (no. TIB-71) were from American Type Culture Collection and cultured in DMEM medium supplemented with 10% FBS. Bone marrow derived macrophages (BMDMs) and dendritic cells (BMDCs) were prepared from the femur and tibia of C57BL/6J mice (Jackson Laboratory) according to the previously published protocols (Xu et al., 2007; Wang et al., 2013, 2016; Ying et al., 2013). Briefly, mice were euthanized by CO_2_ inhalation, then tibias and femurs were removed under sterile conditions, and the bone marrow was flushed out of the cavity by using a 5-ml syringe. After lysis of red blood cells, the resultant cells were resuspended and cultured in RPMI-1640 medium supplemented with 10% FBS, 20% filtered L-929 cell culture supernatant or 10 ng/ml M-CSF, and 50 μg/ml penicillin/streptomycin to generate BMDMs as described previously (Wang et al., 2013; Ying et al., 2013). Culture medium was replenished on day 3 and day 5, and BMDMs were fully differentiated on day 7. To generate BMDCs, GM-CSF and IL-4 were added into the medium to the final concentrations of 20 and 10 ng/ml, respectively. MG-CSF and IL-4 were replenished on days 3 and 5. On day 7, the semi-suspended and loosely-adherent cells were collected and pooled and used as BMDCs as described (Xu et al., 2007; Wang et al., 2013, 2016). Primary mAECs, BMDMs and BMDCs were infected with H1N1 PR/8/34, H3N2 (x:31) A/Aichi/68, or H3N2 A/Hong Kong/8/68 strains (Charles River) at an appropriate multiplicity of infection (MOI) or treated with various agonists as we described previously (Gan et al., 2015, 2016). Briefly, cells were washed once with RPMI-1640, infected with IAV for 1 h at 37°C in basal epithelial cell medium for mAECs or RPMI-1640 for BMDMs and BMDCs, then incubated in epithelial cell growth medium for mAECs or RPMI-1640 with 2.5% FBS for BMDMs and BMDCs without aspirating the viruses for 1 h to facilitate IAV entry into cells. The media were then aspirated and the cells were cultured for 24 h in basal epithelial cell medium for mAECs or RPMI-1640 with 2.5% FBS for BMDMs. For BMDCs, the cells were kept cultured in RPMI-1640 with 2.5% FBS for 24 h.

### Cell Viability Assay

Primary mAECs, BMDMs or BMDCs were seeded into 48-well plates, grown to subconfluence, infected with H1N1 PR8/34 strain (Charles River) at 5 MOI, then incubated in the presence of DMSO, apoptosis or necroptosis pathway inhibitors for 24 or 48 h. Cell viability was assessed by MTS assay using CellTiter A_Queous_ one solution reagent according to the manufacturer's instructions (Promega, Madison, WI). Cell survival rate was calculated by comparison to DMSO-treated control cells and are presented as means ± SE (*n* = 3).

### Western Blot Analysis

Western blot analysis was performed essentially as we described previously (Tang et al., 2000). The membrane was probed with various primary antibodies as indicated and detected with horseradish peroxidase-conjugated secondary antibodies and Bio-Rad Clarity Western ECL substrate (Hercules, CA). Relative changes in the expression levels of interested proteins were measured by densitometric analysis using ImageJ 1.47 software and normalized to actin and are represented as fold of control.

### Statistical Analysis

Data are expressed as means ± SE. Statistical analyses were performed using Microsoft Excel and GraphPad Prism (GraphPad Software, La Jolla, CA). Data were analyzed by Student's *t*-test. *p* < 0.05 was considered statistically significant.

## Results

### IAV Infection Induces ZBP1 Expression and Necroptosis in Mouse Lungs

**Figure 1:**
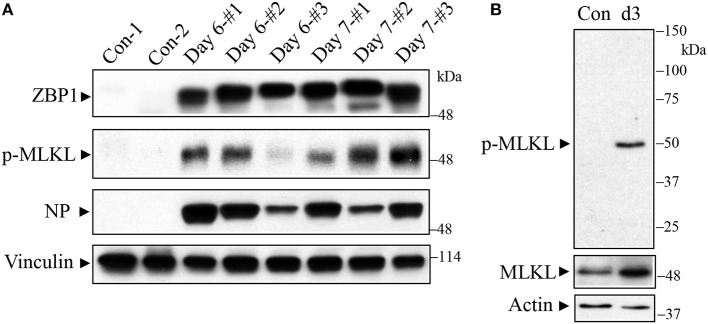
Western blot detection of ZBP1 and necroptosis in IAV-infected mouse lungs. C57BL/6J mice were infected intranasally with a lethal dose (1,000 pfu/mouse, ~2 LD_50_) of the mouse-adapted IAV H1N1 PR/8/34 strain or treated with saline as a negative control (Con). **(A)** Mouse lungs were harvested on days 6 and 7 post-infection and lung homogenates at equal protein amounts were subjected to Western blot analysis with indicated antibodies. **(B)** Bronchoalveolar lavage cells from control and IAV-infected mice on day 3 (d3) post-infection were collected and equal amounts of cell lysates were subjected to Western blot analysis with indicated antibodies. Results represent the findings of three independent experiments.

C57BL/6J mice were infected with a lethal dose of the mouse-adapted IAV H1N1 PR/8/34 strain and lungs were harvested on days 6 and 7 post-infection for Western blot analysis. As shown in Figure 1 (top panel), we found that ZBP1 (Kuriakose and Kanneganti, 2018), a key signaling molecule for IAV-induced cell death, was strongly induced in mouse lungs at both days 6 and 7 post-infection. Necroptosis is mediated by the central RIPK3-MLKL pathway, and phosphorylation of murine MLKL at Ser345 or human MLKL at Ser358 by RIPK3 is a key step in the execution of necroptosis, causing cell membrane disruption (Cho et al., 2009; He et al., 2009; Sun et al., 2012; Zhao et al., 2012; Wang et al., 2014; Rodriguez et al., 2016). We assessed the IAV-induced necroptosis in mouse lung by using an antibody against the phosphorylated-MLKL at Ser345. The specificity of this phospho-specific antibody has been demonstrated in MLKL deficient mouse embryonic fibroblasts by using Western blotting and immunofluorescence microscopy (Rodriguez et al., 2016). However, this antibody has not yet been tested in MLKL deficient mouse tissue sections, so that the specificity of this phospho-antibody has not been demonstrated in such tissues to date. We found that the phosphorylation of MLKL (p-MLKL) at Ser345 was markedly induced by IAV infection in mouse lungs on days 6 and 7 post-infection (Figure 1, 2nd panel). IAV infection in the lungs was confirmed by the detection of NP protein (Figure 1, 3rd panel). Figure 1B shows that a single protein band corresponding to MLKL molecular mass was detected by the p-MLKL antibody in bronchoalveolar lavage cells from IAV-infected but not control mouse lungs. Collectively, these results indicate that IAV infection induces ZBP1 expression and necroptosis in moue lungs.

### Induction of ZBP1 Protein by IAV and Inflammatory Mediators *in vitro* and *in vivo*

**Figure 2:**
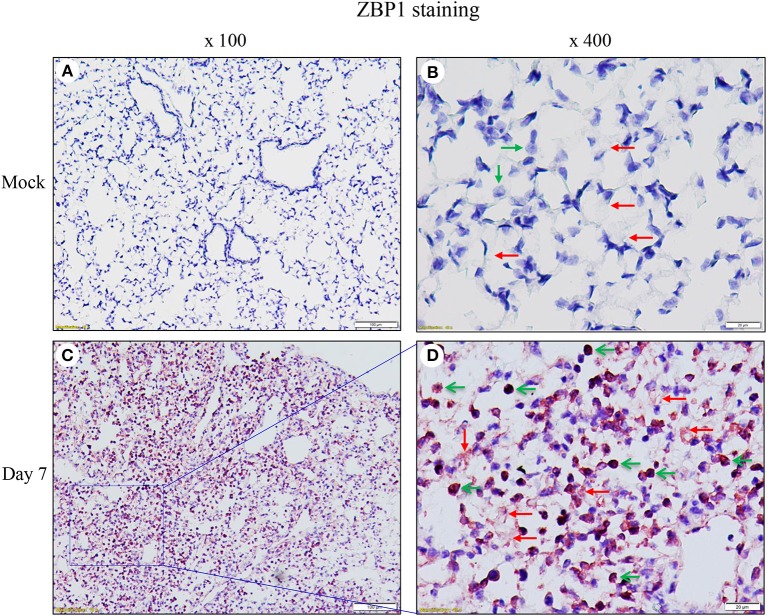
Immunohistochemical detection of ZBP1 in IAV-infected mouse lungs. Lung sections from saline-treated (Mock, **A**,**B**) or H1N1 PR8-infected mice on day 7 post-infection **(C,D)** were subjected to immunostaining with a ZBP1 monoclonal antibody by using Vector M.O.M immunodetection kits (*n* = 5). Positive immunoreactivities (red) were detected in the alveolar epithelial cells (red arrows) and immune cells (green arrows). The region indicated in **(C)** (magnification, x100) is shown at higher magnification in **(D)** (x400).

**Figure 3:**
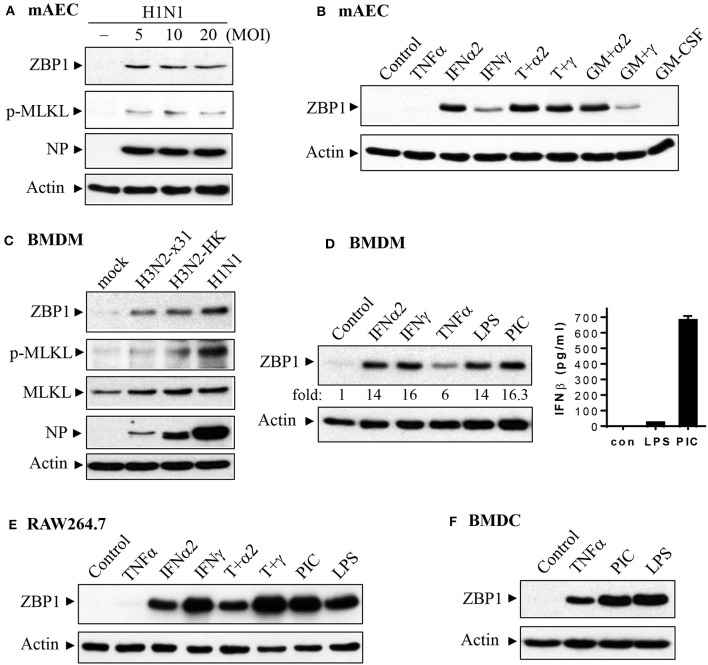
Induction of ZBP1 by IAV infection and immune stimuli in primary mouse alveolar epithelial cells and immune cells. **(A,B)** Primary mAECs were treated with control PBS (- or control), infected with active H1N1 PR/8/34 strain at MOI of 5, 10, or 20, or treated with mouse TNFα (20 ng/ml), IFNα2 (50 ng/ml), IFNγ (50 ng/ml), TNFα plus IFNα2 (T+α2, 20 + 50 ng/ml), TNFα plus IFNγ (T+γ, 20 + 50 ng/ml), GM-CSF (30 ng/ml), GM-CSF plus IFNα2 (GM+α2, 30 + 50 ng/ml), GM-CSF plus IFNγ (GM+γ, 30 + 50 ng/ml) for 24 h. **(C–F)** BMDMs, BMDCs and RAW264.7 murine macrophages were treated with control PBS (mock or control), infected with H1N1 PR/8/34, H3N2 A/Hong Kong/8/68 (H3N2-HK), or H3N2 (x:31) A/Aichi/68 strains at MOI of 5, or treated with mouse IFNα2 (50 ng/ml), IFNγ (50 ng/ml), TNFα (20 ng/ml), LPS (100 ng/ml), poly(I:C) (PIC, 1 μg/ml), TNFα plus IFNα2 (T+α2, 20 + 50 ng/ml), or TNFα plus IFNγ (T+γ, 20 + 50 ng/ml) for 24 h. Equal amounts of cell lysates from **(A–F)** were subjected to Western blotting with indicated antibodies. Results represent the findings of three independent experiments. The production of IFNβ by 24 h treatment of LPS (100 ng/ml) and poly(I:C) (PIC, 1 μg/ml) was shown in the bar graph of **(D)** (*n* = 3).

We performed immunohistochemistry analysis to assess ZBP1 expression in mouse lungs on day 7 post IAV infection. We found that ZBP1 was undetectable in control mouse lung tissues (Figures 2A,B), but was markedly induced by IAV infection in both alveolar epithelial cells (red arrows) and infiltrated immune cells (green arrows) (Figures 2C,D). We next determined the effects of IAV and inflammatory mediators on ZBP1 expression in primary mAECs, BMDMs, and BMDCs *in vitro*. In primary mAECs, ZBP1 could be induced by H1N1 24 h post-infection and H1N1 infection at MOI of 5 was enough for ZBP1 induction, which correlated with the IAV-induced p-MLKL at Ser345 (Figure 3A). ZBP1 was also induced by both IFNα2 and IFNγ, but the effect of IFNα2 was much higher than IFNγ (Figure 3B). TNFα plus IFNγ but not IFNα2 synergistically induced ZBP1 expression in mAECs although TNFα alone had a very minor effect. In addition, GM-CSF alone or with IFNs did not affect ZBP1 expression. In BMDMs, both ZBP1 expression and MLKL phosphorylation were induced by IAV infection with H1N1 PR/8/34, H3N2 A/Hong Kong/8/68, or H3N2 (X:31) A/Aichi/68 strains; and the induction magnitudes correlated with the cell susceptibility to IAV infection revealed by NP protein levels (Figure 3C). Interestingly, both IFNα2 and IFNγ markedly induced ZBP1 expression in BMDMs, and the effect of IFNγ was slightly higher than IFNα2 (Figure 3D), which is different from those observed in mAECs (Figure 3B). This difference in the sensitivity to IFNs may be due to the difference in receptor expression between mAECs and BMDMs. Furthermore, ZBP1 was readily induced to a comparable level by TLR4 agonist LPS and TLR3 agonist poly(I:C) in BMDMs. It should be noted that in contrast to poly(I:C), LPS only had a minor effect on the production of type-I IFNβ (Figure 3D, bar graph). Similar data for the inflammatory mediator-induced expression of ZBP1 were obtained in a murine macrophage cell line RAW264.7 (Figure 3E). In addition, the synergistic effect of TNFα plus IFNγ on ZBP1 expression was also observed in RAW264.7 macrophages. Lastly, we found that ZBP1 expression was robustly induced by TNFα, poly(I:C), and LPS in BMDCs (Figure 3F). These findings indicate that inflammatory mediators such as IFNs, TLR3, and TLR4 agonists, and TNFα, in addition to IAV, are potent inducers of ZBP1 expression in alveolar epithelial cells and infiltrated monocyte-derived macrophages and dendritic cells.

### IAV Infection Induces Strong Necroptosis in Both Infiltrating Immune and Lung Epithelial Cells *in vivo*

**Figure 4:**
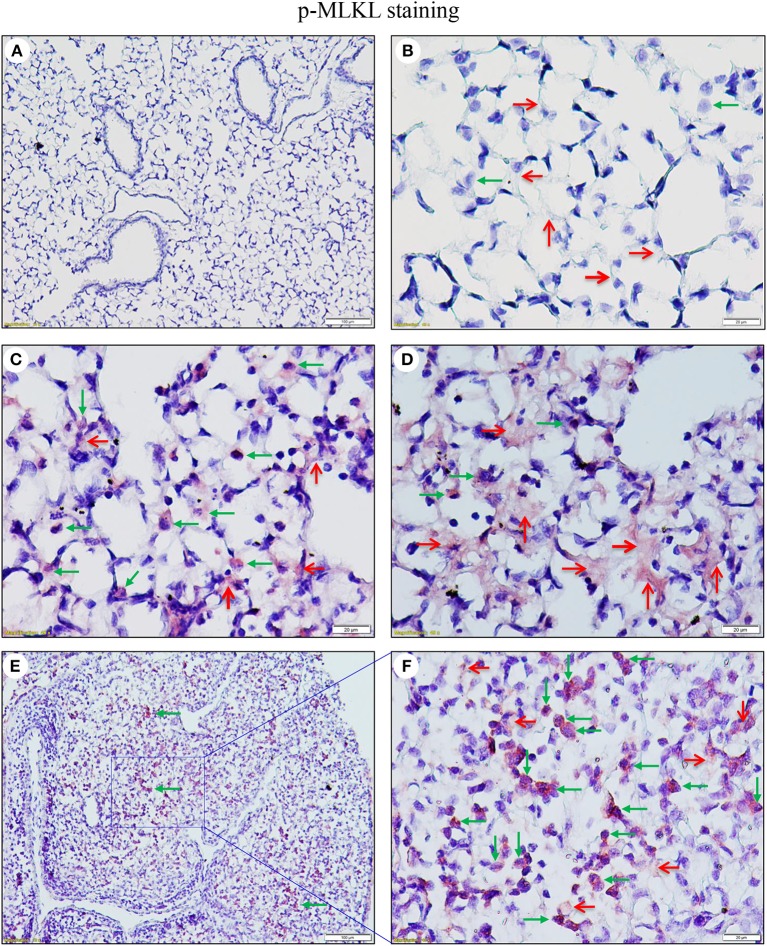
IAV induces phosphorylation of MLKL in alveolar epithelial cells and infiltrated immune cells in infected mouse lungs. Lung sections from saline-treated (Mock, **A**,**B**) or PR8-infected mice on day 7 post-infection **(C–F)** were subjected to immunostaining with a phospho-MLKL (Ser345) monoclonal antibody using a Vector M.O.M immunodetection kit (*n* = 6). Positive p-MLKL immunoreactivities (red) were detected in the alveolar epithelial cells (red arrows) and immune cells (green arrows). The region indicated in **(E)** (magnification, x100) is shown at higher magnification in **(F)** (x400). Final magnification for **(A)** is x100 and for **(B–D)** x400. In **(A–D)**, the lung sections were from inflated mouse lungs by intratracheal infusion of 10% formalin to total lung capacity.

**Figure 5:**
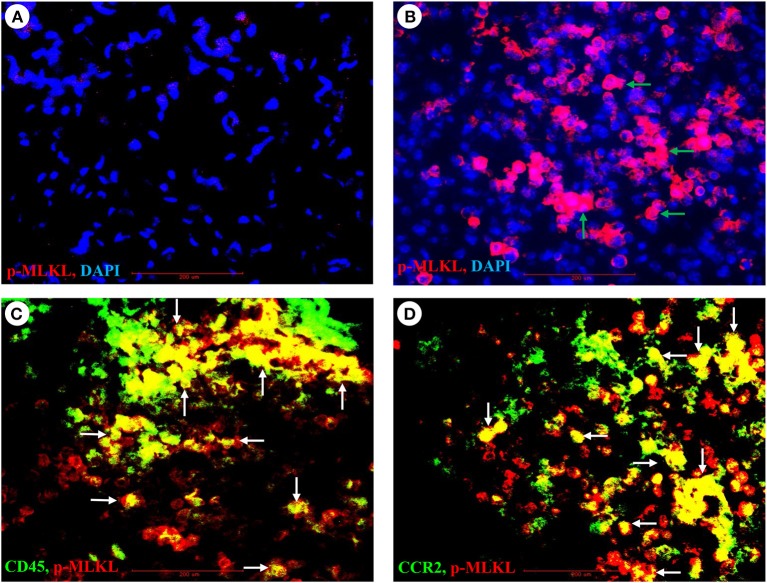
Immunofluorescence detection of IAV-induced phosphorylation of MLKL and its co-localization with pulmonary infiltrated immune cells. **(A,B)** Lung sections from control **(A)** or PR8-infected mice **(B)** on day 7 post-infection were subjected to immunofluorescence staining with a phospho-MLKL (Ser345) monoclonal antibody by using Vector M.O.M immunodetection kit followed by Alexa Fluor 568-labeled goat anti-mouse secondary IgG (*n* = 6). Positive p-MLKL immunoreactivities are stained red (indicated by green arrows) and cell nuclei stained blue by DAPI. Final magnification is x400. **(C,D)** Lung sections from PR8-infected mice on day 7 post-infection were subjected to double immunofluorescence staining with the phospho-MLKL (Ser345) mouse monoclonal antibody, Alexa Fluor-488 conjugated CD45 or FITC conjugated CD192/CCR2 rat monoclonal antibodies as described in section Materials and Methods. Positive p-MLKL immunoreactivities are stained red, and CD45 or CCR2 are stained green. The co-localization or overlay is shown as yellow and indicated by white arrows. Final magnification: x400 (*n* = 6).

As necroptosis was induced by IAV infection in mouse lungs on days 6 and 7 post-infection (Figure 1), we performed immunohistochemistry and immunofluorescence analyses to characterize necroptosis in mouse lungs on day 7 post IAV infection. As shown in Figure 4, the p-MLKL at Ser345 was apparently induced by IAV infection in both alveolar epithelial cells (red arrows) and immune cells (green arrows). Figure 4D shows that p-MLKL immunoreactivities were clearly observed in the spread and thin squamous type I alveolar epithelial cells and in immune cells. In many lesion areas, p-MLKL immunostaining signals appeared to be stronger in immune cells compared with alveolar epithelial cells (Figures 4C,F). Moreover, immunofluorescence analysis revealed that the p-MLKL was strongly induced by IAV infection (Figures 5A,B), confirming the occurrence of necroptosis in mouse lungs. Double immunofluorescence analysis showed that over 50% of p-MLKL positive cells were overlaid with biomarkers for hematopoietic immune cells (CD45 and CCR2; Figures 5C,D). Collectively, these findings indicate that IAV infection induces strong necroptosis in infiltrated immune cells and lung structural alveolar epithelial cells *in vivo* on day 7 post-infection.

### Cell Type-Specific Responses to IAV-Induced Cell Death Upon Inhibition of Caspases and/or RIPK3

**Figure 6:**
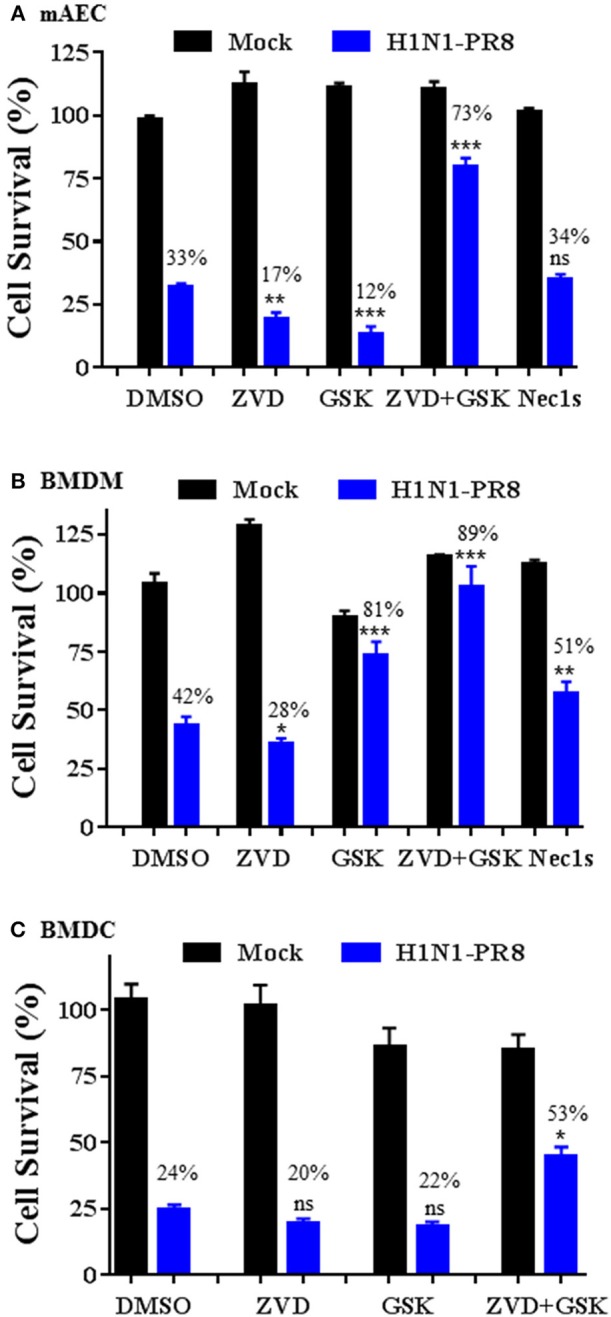
Cell type-specific responses to IAV-induced cell death upon inhibition of caspases and/or RIPK3. Primary mAECs, BMDMs, and BMDCs were treated with control PBS (Mock) or infected with H1N1 PR8 at 5 MOI in the presence of DMSO, v-ZVD-FMK (ZVD, 60 μM), GSK872 (GSK, 5 μM), v-ZVD-FMK plus GSK872 (ZVD+GSK), or RIPK1 inhibitor II (Nec-1s, 5 μM) for 24 h **(B,C)** or 48 h **(A)**. Cell viability was assessed by MTS assay (CellTiter A_queous_ One Solution Assay) and cell survival rates were calculated by comparison to DMSO-treated control mock cells and are presented as means ± SE (*n* = 3). NS, no significance; *p < 0.05; **p < 0.01; ***p < 0.001 vs. DMSO. Cell survival rates relative to individual mock control groups were also shown over the bar as indicated.

We assessed the involvement of apoptosis and necroptosis pathways to IAV-induced cell death in primary mAECs, BMDMs, and BMDCs. As shown in Figure 6A, H1N1 PR8 induced cell death in primary mAECs and the effect was enhanced by co-treatment with a pan-caspase inhibitor v-ZVD-FMK (Slee et al., 1996). Similarly, inhibition of the necroptosis key mediator RIPK3 with GSK872 (Kaiser et al., 2013), but not of RIPK1 with Nec-1s (Takahashi et al., 2012), enhanced IAV-induced cell death. However, the IAV-induced cell death was markedly and significantly prevented by v-ZVD-FMK plus GSK872, indicating that necroptosis and caspase-dependent apoptosis and/or pyroptosis are involved in IAV-induced cell death in primary mAECs. On the other hand, the IAV-induced cell death in BMDMs was significantly inhibited by GSK872, Nec-1s, and v-ZVD-FMK plus GSK872; although cell death was enhanced by v-ZVD-FMK (Figure 6B). In BMDCs, we found that the IAV-induced cell death was neither inhibited by v-ZVD-FMK nor GSK872, but was significantly prevented by v-ZVD-FMK plus GSK872 (Figure 6C). Collectively, these findings indicate that necroptosis and caspase-dependent apoptosis and/or pyroptosis are involved in IAV-induced cell death in primary mAECs, BMDMs, and BMDCs, and that different cell death signaling mechanisms may be operative in different types of cells in lungs.

## Discussion

Programmed cell death, especially necroptosis has emerged as an important mechanism in the pathogenesis of influenza (Atkin-Smith et al., 2018; Downey et al., 2018; Fujikura and Miyazaki, 2018). The central model of necroptosis involves RIPK3 and the RIPK3-mediated p-MLKL (Cho et al., 2009; He et al., 2009; Sun et al., 2012; Zhao et al., 2012; Wang et al., 2014; Silke et al., 2015; Wallach et al., 2016; He and Wang, 2018). Recent studies in murine models have identified ZBP1 (also known as DAI/DLM-1) as a key mediator of IAV-induced necroptosis through its interaction with the downstream effectors RIPK3 and MLKL (Kuriakose et al., 2016; Thapa et al., 2016; Kesavardhana et al., 2017; Kuriakose and Kanneganti, 2018). ZBP1 also mediates other forms of programmed cell death including apoptosis and/or pyroptosis independent of RIPK3 kinase activity. It has been shown that ZBP1- or RIPK3-deficient mice have high lung virus titers and are hyper-susceptible to lethal IAV H1N1 infection (Nogusa et al., 2016; Thapa et al., 2016; Downey et al., 2017). The data are in agreement with the hypothesis that programmed cell death is a host defense mechanism that effectively eliminates the replicative niche of the virus (Atkin-Smith et al., 2018; Downey et al., 2018; Fujikura and Miyazaki, 2018). By contrast, another group reported that ZBP1 deficiency protected mice from lung epithelial damage and mortality by IAV H1N1 infection, despite having higher lung virus titers and slower recovery times than controls (Kuriakose et al., 2016). Moreover, RIPK3 knockout protects cIAP2-deficient mice from lethal IAV H1N1 infection via maintenance of pulmonary tissue homeostasis and mice from influenza H7N9 infection via inhibition of host inflammatory response, respectively (Rodrigue-Gervais et al., 2014; Xu et al., 2017). These findings support the hypothesis that ZBP1 or RIPK3 deficiency protects against cell death, preserves alveolar epithelial cell integrity and controls pulmonary inflammation during severe IAV infection. Hence, programmed cell death could be a double-edged sword for IAV infection. While programmed cell death effectively eliminates virus replication, aberrant or uncontrolled cell death could cause pulmonary dysfunction and inflammation; and thereby contribute to morbidity and mortality depending on the severity of infection and host cell responses (Herold et al., 2015; Fujikura and Miyazaki, 2018). Thus, delineation of the mechanisms participating in these responses and the responses of resident and infiltrating cells assumes importance.

The present study provides new direct evidence that IAV infection induces necroptosis in infiltrating immune cells and lung structural alveolar epithelial cells *in vivo*. Unlike apoptosis, necroptosis causes cell membrane rupture and the release of damage-associated molecular patterns and is a strong trigger of innate and adaptive immune responses (Silke et al., 2015; Wallach et al., 2016; He and Wang, 2018). The strong necroptosis occurring in infiltrated immune cells may be a critical mechanism in the pathogenesis of influenza. Whether necroptosis favors host antiviral immunity or causes lung inflammation and damage await the generation of mouse models with cell type-specific knockout of RIPK3 or ZBP1. We posit that deeper understanding of the IAV-induced cell type-specific necroptosis and their regulation may offer a novel strategy to control host immune response and lung epithelial cell damage during severe IAV infection.

During IAV infection, ZBP1 binds to IAV genomic RNA and the activated ZBP1 then associates with RIPK3 via RHIM domains and triggers programmed cell death via necroptosis, apoptosis, and/or pyroptosis in IAV-infected cells (Kuriakose et al., 2016; Thapa et al., 2016; Kesavardhana et al., 2017). We found that ZBP1 was barely detectable in control mouse lungs, mAECs and BMDCs, but was slightly expressed in control BMDMs. It has been shown that IAV-induced expression of ZBP1 is mediated by type-I IFN in BMDMs *in vitro* (Kuriakose et al., 2016). Recently, IFN regulatory factor-1 was identified as a transcriptional regulator of ZBP1, upregulating ZBP1 expression independently of type I IFN production and signaling in BMDMs (Kuriakose et al., 2018). In addition to IAV and type-I IFN, we found that ZBP1 was markedly induced by type-II IFNγ, TLR3 agonist poly(I:C), and TLR4 agonist LPS in primary mAECs, BMDMs, and BMDCs. Moreover, TNFα plus IFNγ but not IFNα2 synergistically induced ZBP1 expression in the cells. Interestingly, both LPS (100 ng/ml) and poly(I:C) (1 μg/ml) induced ZBP1 expression at a comparable level although LPS only had a minor effect on the production of type-I IFNβ compared with poly(I:C). Whether an IFN-independent mechanism is involved in ZBP1 induction by LPS merits further investigation. Type-1 and type-II IFNs, TLR3 and TLR4 agonists, and TNFα are critical inflammatory mediators implicated in the pathogenesis of IAV infection (Van Reeth, 2000; Herold et al., 2015; Prantner et al., 2017). It should be noted that the induction of ZBP1 by these inflammatory mediators is apparently higher than that caused by IAV infection. In IAV-infected mouse lungs, we found that ZBP1 was markedly induced in both alveolar epithelial cells and immune cells. Based on our findings, the strong induction of ZBP1 in IAV-infected lungs may largely result from the effects of pro-inflammatory mediators in lung tissues. As ZBP1 is a key mediator of IAV-induced programmed cell death, targeting pro-inflammatory mediators or host immune response may offer an effective strategy to control cell death and lung epithelial damage during severe IAV infection.

IAV-induced programmed cell death has been studied in mouse fibroblasts (Kuriakose et al., 2016; Nogusa et al., 2016), a murine lung epithelial type I cell line (LET1) (Nogusa et al., 2016), and BMDMs (Kuriakose et al., 2016), but not in primary mAECs which are the major type of cells infected during IAV infection. In agreement with LET1 cells but not fibroblasts, we found that H1N1 PR8-induced cell death in primary mAECs was significantly enhanced by co-treatment with a pan-caspase inhibitor v-ZVD-FMK (Slee et al., 1996), suggesting a switch to and activation of the necroptosis pathway. Similarly, inhibition of the kinase activity of necroptosis key mediator RIPK3 with GSK872 (Kaiser et al., 2013) also significantly enhanced the IAV-induced cell death in primary mAECs, suggesting a switch to and activation of apoptosis and/or pyrotopsis pathways. These findings oppose the observations in murine fibroblasts (Nogusa et al., 2016) and indicate that IAV-induced cell death signaling pathways could differ between primary mAECs and fibroblasts, although the IAV-induced cell death in both cell types was markedly prevented by v-ZVD-FMK plus GSK872. In BMDMs, we found that the IAV-induced cell death could be significantly prevented by RIPK3 inhibitor GSK872 or RIPK1 inhibitor Nec1s alone although the cell death was enhanced by co-treatment with v-ZVD-FMK. In BMDCs, the IAV-induced cell death was neither inhibited by v-ZVD-FMK nor GSK872, but was significantly prevented by v-ZVD-FMK plus GSK872. Hence, our findings indicate that necroptosis and caspase-dependent apoptosis and/or pyroptosis are involved in IAV-induced cell death in primary mAECs, BMDMs, and BMDCs. The underlying cell death signaling mechanisms and regulation may operate differently in different cell types. GSK872, the specific inhibitor of RIPK3, can be used to prevent IAV-induced cell death (necroptosis) in BMDMs, but not in BMDCs and mAECs.

In summary, we provide *in vivo* evidence showing that IAV infection induces ZBP1 expression in mouse lungs and a strong necroptosis in infiltrating immune cells and alveolar epithelial cells. We further show that ZBP1 can be strongly induced by inflammatory mediators highly implicated in the pathogenesis of influenza infection, including IFNs, TLR3, and TLR4 agonists, and TNFα; and provide evidence suggesting that different cell death signaling mechanisms are operative in different types of cells in lungs during IAV infection.

## Data Availability

All datasets generated for this study are included in the manuscript and/or the supplementary files.

## Ethics Statement

All animal experiments were approved by the Institutional Animal Care and Use Committee at the University of Texas Health Science Center at Tyler.

## Author Contributions

YW, QH, and HT conceived and designed the study. YW, QH, JF, B-GJ, and HT performed the experiments and analyzed the data. HT wrote and edited the manuscript. AK, BS, and SI provided materials and advice on data interpretation and edited the manuscript.

### Conflict of Interest Statement

The authors declare that the research was conducted in the absence of any commercial or financial relationships that could be construed as a potential conflict of interest.

## Abbreviations

BMDCs: bone marrow derived dendritic cells
BMDMs: bone marrow derived macrophages
mAECs: mouse alveolar epithelial cells.
